# Concomitant diagnosis of bipolar disorder and tuberous sclerosis - a case report

**DOI:** 10.1192/j.eurpsy.2022.2278

**Published:** 2022-09-01

**Authors:** M. Santos, T. Ferreira, P. Neves, J. Peres

**Affiliations:** 1 Hospital Prof. Doutor Fernando Fonseca, Psychiatry, Amadora, Portugal; 2 Hospital Prof Doutor Fernando Fonseca, Mental Health Department, Amadora, Portugal; 3 Hospital Prof Doutor Fernando Fonseca, Amadora, Portugal, Neurology, Amadora, Portugal

**Keywords:** Tuberous sclerosis, Neuropsychiatry, bipolar disorder

## Abstract

**Introduction:**

Tuberous sclerosis is a multisystem genetic disorder. It is associated with significant psychiatric comorbidity mainly autistic disorders, hyperkinetic disorders, depression and anxiety. It is rarely associated with psychosis and bipolar disorder.

**Objectives:**

To describe the case of a 34-year-old male with concomitant diagnosis of bipolar disorder and tuberous sclerosis.

**Methods:**

Case report based on clinical records. Brief literature review using articles searched in the PubMed/MEDLINE database using the terms “tuberous sclerosis”, “bipolar disorder” and “neuropsychiatric”.

**Results:**

The patient presented at our Emergency Department 3 years ago with a mixed episode with psychotic symptoms with 1 month of duration. Prior history of two hypomanic episodes, but no depressive episodes. High baseline functionality. Sporadic use of alcohol and cannabis. No family history of psychiatric or neurological diseases. Diagnostic work-up showed no relevant results, aside from small calcifications in brain CT. He was admitted to our ward and medicated with aripiprazol (titrated up to 30 mg), leading to full remission of the clinical picture. The patient was referred to our outpatient clinic and stayed with medication for 1,5 years. One year after, he presented a sudden episode of mutism and perplexity with quick remission. The EEG wielded no relevant results. New brain CT showed signs of tuberous sclerosis. He was referred to Neurology and subsequent assessments, including brain MRI, led to the fulfilment of clinical criteria for tuberous sclerosis.

**Conclusions:**

This case illustrates the possibility of concomitant diagnosis of bipolar disorder and tuberous sclerosis. The possible association between these disorders is discussed.

**Disclosure:**

No significant relationships.

